# SSTR2 Mediates the Inhibitory Effect of SST/CST on Lipolysis in Chicken Adipose Tissue

**DOI:** 10.3390/ani14071034

**Published:** 2024-03-28

**Authors:** Xiao Zhang, Jiannan Zhang, Tianjiao Huang, Xinglong Wang, Jiancheng Su, Jiliang He, Ningkun Shi, Yajun Wang, Juan Li

**Affiliations:** 1 Key Laboratory of Bio-Resources and Eco-Environment of Ministry of Education, College of Life Sciences, Sichuan University, Chengdu 610017, China; 2 Animal Disease Prevention and Food Safety Key Laboratory of Sichuan Province, College of Life Sciences, Sichuan University, Chengdu 610017, China

**Keywords:** chicken, adipose tissue, SSTR2, lipolysis

## Abstract

**Simple Summary:**

The chicken is one of the model organisms of birds and is important for the poultry industry. Chickens have many unique metabolic characteristics, and the metabolic regulatory mechanisms for these characteristics remain poorly understood. For example, somatostatin has been reported to have an anti-lipolytic effect but its mediator remains to be identified. In our study, we found that somatostatin receptor 2 (SSTR2) is highly expressed in chicken adipose tissue, and SSTR2 antagonists can block the anti-lipolytic effect of somatostatin, supporting that this effect is mediated by SSTR2. At the same time, we found that SST28 can stimulate the proliferation of chicken preadipocytes, and this proliferation effect may be mediated by SSTR2 through the MAPK/ERK signaling pathway. The present study identified the primary mediator responsible for the anti-lipolytic effect of somatostatin in chickens, thereby reinforcing its significant role in adipose metabolism.

**Abstract:**

Somatostatin shows an anti-lipolytic effect in both chickens and ducks. However, its molecular mediator remains to be identified. Here, we report that somatostatin type 2 receptor (SSTR2) is expressed at a high level in chicken adipose tissue. In cultured chicken adipose tissue, the inhibition of glucagon-stimulated lipolysis by somatostatin was blocked by an SSTR2 antagonist (CYN-154086), supporting an SSTR2-mediated anti-lipolytic effect. Furthermore, a significant pro-proliferative effect was detected in SST28-treated immortalized chicken preadipocytes (ICP-1), and this cell proliferative effect may be mediated through the MAPK/ERK signaling pathway activated by SSTR2. In summary, our results demonstrate that SSTR2 may regulate adipose tissue development by affecting the number and volume of adipocytes in chickens.

## 1. Introduction

Somatostatin is a 14-amino acid polypeptide (SST14) first identified in the ovine hypothalamus [1]. Later, a 28-amino acid peptide (SST28), which is an N-terminally extended form of SST14, was discovered in the porcine intestinal tract [2]. Further studies proved that SST14 and SST28 are bioactive peptides processed from the same somatostatin precursor in mammals, playing important roles in behavior, cognition, endocrine system, exocrine system, immunity, cell proliferation, and smooth muscle contraction. Recently, a novel polypeptide named cortistatin (CST) has been identified in multiple species including humans, mice, rats, chickens, zebrafish and frogs [3,4,5,6,7]. CSTs, exhibiting a structure highly similar to SST14 and SST28, have been regarded as analogues of SSTs, playing a role in central nervous system (CNS) development and pituitary differentiation [8,9].

SST and CST have been reported to exert various physiological effects by binding to their receptors (SSTRs) [10]. So far, five somatostatin receptors have been found, all of which are G-protein-coupled receptors [10,11]. Among the receptors, SSTR1-4 show equal affinity with SST14 and SST28, whereas SSTR5 shows a different binding affinity with SST14 (EC_50_: 2.24 ± 0.36 nM) and SST28 (EC_50_: 0.19 ± 0.03 nM) [12]. Activation of these receptors is reported to couple with Gi proteins to inhibit adenylate cyclase activity [13,14]. Although these five receptors have similar structures and functions, they are expressed in different tissues and mediate diverse physiological effects [10,15,16]. For example, in primary cultured human fetal pituitary cells, both SSTR2 and SSTR5 mediate the inhibition of GH and TSH release, whereas only SSTR2 mediates the inhibition of prolactin release [17]. In humans and mice, SSTR2 expressed in pancreatic α cells is involved in the inhibition of glucagon (GCG) secretion, and SSTR5 expressed in pancreatic β cells is involved in the inhibition of insulin secretion [16,18]. SSTR2 has been reported to be involved in the inhibition of bile acid secretion in cultured mouse cholangiocytes [19]. Uncovering the tissue expression preference and isoform functional specificity of SSTRs is an open and important matter for scientific researchers.

Five chicken somatostatin receptors (*SSTR1-5*) have been cloned from the brain [20]. All the cSSTRs have been revealed to be functionally coupled with Gi proteins. Similar to mammals, chicken SSTR1-4 are efficiently activated in vitro by CST14, SST14 and SST28, whereas SSTR5 only binds SST28 with nanomolar affinity [20]. Different from the reports in mammals, inhibition of chicken pituitary GH secretion is mediated by SSTR2, whereas inhibition of TSH secretion is mediated by SSTR2 and SSTR5 [21,22].

In isolated chicken adipocytes, Strosser et al. have found that SST14 and SST28 inhibit glucagon-stimulated glycerol release [23]. Glucagon is the main lipolytic hormone in domestic chickens and transmits signals through the glucagon receptor (GCGR) [24,25,26,27]. In pancreatectomized or hypophysectomized ducks, somatostatin infusion resulted in a decrease in plasma-free fatty acid, suggesting a direct anti-lipolytic effect of somatostatin on adipose tissue [28]. Although the anti-lipolytic effect of SST in birds is clear, its direct mediator remains to be identified. This study comprehensively investigated the distribution of SSTRs in chicken tissue through transcriptome data and found that *SSTR2* is abundantly expressed in chicken adipose tissue. In addition, the effect of SST28 on chicken preadipocyte proliferation was also explored. The chicken is one of the model organisms of birds. The results of this study provide basic data for analyzing the regulatory mechanism of lipid metabolism in birds.

## 2. Materials and Methods

### 2.1. Chemicals, Primers, Peptides and Antibodies

Chicken SST14, SST28 and CST14 were synthesized by GL Biochem Ltd. (Shanghai, China). The purity of synthesized peptides was more than 95% (analyzed by HPLC) and their structures were verified by mass spectrometry. A goat IgG-Immunohistochemistry Kit (SA1023) was purchased from Boster Biological Technology Co., Ltd (Wuhan, China). Rabbit anti-human SSTR2 polyclonal antibody was purchased from Abcam technology (Cambridge, UK). Antibodies of ERK1/2 and phosphorylated ERK1/2 were purchased from Cell Signaling Technology, Inc. (Danvers, MA, USA).

### 2.2. Animals

The Lohmann White Layer chickens (one-year-old and four-month-old) used in this study were purchased from a local commercial company. The purchased chickens were transported a short distance to the laboratory and raised in the laboratory’s animal room where the chickens were allowed to eat and drink ad libitum with a normal light cycle. To avoid the impact of transportation stress, the purchased chickens were raised in the animal room for at least one week before sampling.

### 2.3. Tissues Collection

Four male chickens (one-year-old, Lohmann White Layer) in good physiological condition were sacrificed, and various tissue (clavicular fat, cardiac fat, abdominal fat, subcutaneous fat, liver and muscle) was collected, frozen in liquid nitrogen and stored at −80 °C for total RNA extraction. Total RNA was extracted from chicken tissues using RNAzol (Molecular Research Center, Cincinnati, OH, USA) according to the manufacturer’s instructions and dissolved in diethylpyrocarbonate-treated H_2_O.

### 2.4. RT-PCR and Quantitative Real-Time PCR Assays

For reverse transcription, 2 μg of total RNA and 0.5 μg of oligodeoxythymide were mixed in a total volume of 5 μL, incubated at 70 °C for 10 min and cooled at 42 °C for 2 min. Then, the first-strand buffer, 0.5 mM each deoxynucleotide triphosphate (dNTP), and 100 U Moloney murine leukemia virus (MMLV) reverse transcriptase (Takara, Japanese) were added into the reaction mix in a total volume of 10 μL. Reverse transcription (RT) was performed at 42 °C for 90 min.

According to our previously established method [29], quantitative real-time PCR was performed to examine the expression of *SSTR2* mRNA among chicken tissues. The qPCR primers of chicken *SSTR2* (Sense: 5′-AGCGCGGAGCCGATCGGAACG-3′; Antisense: 5′-CTGGGCCGTCTCCACTTGGCAG-3′) were designed based on the sequence in the GenBank (NM_001030345.3). The primers (20 μM), dNTP (10 mM), KOD Buffer, KOD FX polymerase (TOYOBO, Japanese), Eva Green (Biotium, CA, USA), MilliQ-H_2_O and templates were mixed in a total volume of 20 μL. Amplification of *SSTR2* fragments was performed on the CFX96 Real-time PCR Detection System (Bio-Rad, Hercules, CA, USA). The amplification conditions included an initial denaturation for 10 min at 94 °C followed by 20 s denaturation at 94 °C, 15 s annealing at 60 °C, and 30 s extension at 72 °C for 40 cycles.

### 2.5. Tissue Expression Analysis of Chicken SSTR2 Using RNA-Seq Data

Multiple RNA-seq datasets were employed for the investigation of the tissue expression of chicken *SSTR2*. These datasets are the combination of our own results and other publications [30,31,32].

The RNA-seq data from Lohmann White was collected from 38 tissues of six one-year-old individuals (three males and three females), including abdominal fat, adrenal gland, bursa of Fabritius, cecum, cerebellum, cerebrum, crop, duodenum, gizzard, heart, hindbrain, hypothalamus, ileum, infundibulum, jejunum, kidney, liver, lung, magnum, midbrain, muscle, ovary, pancreas, parathyroid glands, pineal body, pituitary, proventriculus, rectum, retina, skin, spinal cord, spleen, testis, thymus gland, thyroid gland, tongue, uterus and visceral fat. The data has been published in a preprint and stored in the CNGB data archive (CNSA) of the China National GenBank Data Base (CNGBdb) with the accession number CNP0003404 [32]. The RNA-seq data from the red jungle fowl was published by Burge et al., in their study on the evolutionary dynamics of vertebrates (SRP016501) [30]. This publication contains the transcript information of adipose tissue, adrenal gland, breast muscle, cerebellum, cerebrum, heart, hypothalamus, kidney, liver, lung, ovary, proventriculus, sciatic nerve, spleen, testis and pituitary, which were sampled from a female and a male red jungle fowl. The RNA-seq data from J-line chickens are publicly available and were obtained from 21 tissues of nine hens aged 16–17 weeks by the Roslin Institute, including breast muscle, bursa, caecal tonsil, cerebellum, duodenum, gizzard fat, harderian gland, heart muscle, ileum, kidney, left optic, liver, lung, ovary, pancreas, proventriculus, skin, spleen, thymus, thyroid and trachea (ERP014416). The RNA-seq data of Jingxing-Huang chicken, a native Chinese broiler strain, were sampled from abdominal adipose tissues at different developmental stages (PRJCA001192) [31]. The abdominal fat tissue samples for each developmental stage were taken from three female Jingxing-Huang chickens.

As previously described, this study used conventional transcriptome data analysis pipelines to calculate tissue expression information for *SSTR2* from these raw transcriptome data [32]. Transcripts per million (TPM) values were used to express the relative abundance of *SSTR1-5* transcripts.

### 2.6. Immunohistochemical Staining

To examine the distribution of SSTR2 in chicken tissues, immunohistochemical staining (IHC) was used in our experiment, as described in our previous study [33]. Firstly, the fresh adipose tissue was removed from one-year-old Lohmann White Layer roosters and was fixed with 4% paraformaldehyde for 24 h. The tissue was then embedded in paraffin and cut into 5 μm sections. Subsequently, the paraffin sections were dewaxed, hydrated, and incubated with 0.25% trypsin at 37 °C for 20 min, and then washed in PBS. The samples were further treated with 3% H_2_O_2_ for 20 min at room temperature to irreversibly inactivate the endogenous peroxidase, with PBS wash. These samples were further incubated with 5% bovine serum albumin (BSA) solution for 1 h to block non-specific binding of the antibody. Pre-blocked sections were incubated overnight at 4 °C in PBS containing 5% BSA and diluted (1:200) SSTR2 antibody and washed in PBS. Then, the sections were incubated with biotinylated goat anti-rabbit IgG for 1 h at room temperature before being washed in PBS, and incubated with a streptavidin–biotin complex-horseradish peroxidase Kit (SABC Kit) at room temperature for 30 min according to the manufacturer’s instructions (Boster Biological Technology Co., Ltd., Wuhan, China). Immunoreactive signals were detected using a DAB (3, 30-Diaminobenzidine) substrate Kit (Boster Biological Technology Co., Ltd., Wuhan, China). Sections with the same treatment, but incubated with primary antibody diluent without SSTR2 antibody, were used as a negative control.

### 2.7. Functional Characterization of Chicken SSTR2

According to the cDNA sequences of chicken *SSTR2* (NM_001030345.3), gene-specific primers were designed to amplify the complete open reading frame (ORF) of *SSTR2* from chicken pituitary. The amplified PCR products of *cSSTR2* were cloned into pcDNA3.1 (+) expression vector (Invitrogen, Carlsbad, CA, USA) and sequenced (BGI, Beijing, China).

Chinese hamster ovary (CHO) cells were cultured in 10 cm dishes (NUNC, Rochester, NY, USA) in Dulbecco’s Modified Eagle’s Medium (DMEM) supplemented with 10% (*v/v*) fetal bovine serum (HyClone, Logan, UT, USA), 100 U/mL penicillin G, and 100 g/mL streptomycin (Nunc, Rochester, NY, USA), and incubated at 37 °C with 5% CO_2_.

According to the methods described in our previous study, the functionality and signaling properties of chicken SSTR2 were evaluated in CHO cells by the pGL4-SRE-Luciferase reporter system [20]. Briefly, CHO cells were prepared and seeded into 6-well plates one day before transfection. Then, the cells were transfected with a mixture containing 250 ng of receptor expression plasmid (cSSTR2 or empty pcDNA3.1 vector), 750 ng of pGL4-SRE-Luciferase plasmid, 2 μL of jetPRIME (Polyplus transfection, Illkirch, France) and 200 μL of jetPRIME transfection buffer. After 24 h of transfection, the cells were divided into 96-well plates and cultured for 24 h, and then treated with SST28 at a dose of 10^−12^–10^−6^ M for 6 h. Finally, CHO cells were lysed with 1 × cell culture lysis buffer (Promega, Madison, WI, USA) for luciferase assay, and the luciferase activity of the cell lysate was measured using a multimode microplate reader (TriStar LB941, Berthold Technologies, Bad Wildbad, Germany) according to the manufacturer’s instructions.

### 2.8. Western Blot

As described in our previous study, CHO cells were employed to test whether chicken SSTR2 activation can enhance ERK1/2 (42/44 kDa) phosphorylation [34]. CHO cells were cultured in DMEM supplemented with 10% (*v/v*) fetal bovine serum, 100 U/mL penicillin G and 100 μg/mL streptomycin in a 90 mm dish and incubated at 37 °C with 5% CO_2_. Cells were then plated on a 12-well plate one day before transfection. A mixture containing 500 ng of chicken *SSTR2* expression plasmid and 1 μL of jetPRIME (Polyplus transfection, Strasbourg, France) was prepared in 100 μL of jetPRIME transfection buffer and added to the culture medium. After 24 h of transfection, CHO cells were treated with SST28 (10 nM) for 10 min. After aspiration of the medium, CHO cells were lysed and used for Western blot detection of phosphorylated ERK1/2 (42/44 kDa).

### 2.9. Cell Counting Assay

ICP-1 cells were cultured according to the previous study [35]. These cells were seeded into 6-cm plates and transfected with 2 μg of *SSTR2* expression plasmid the next day. After 24 h of transfection, the cells were plated in a 96-well plate and treated with SST28 for 24 h. Then 10 μL of CCK-8 kit (Servicebio, Wuhan, China) was added to each well to incubate for 1 h, and the absorbance of OD450 was measured using a microplate reader. The medium containing CCK-8 reagent was removed, and complete medium containing SST28 was added and incubated for 24 h. Then, 10 μL CCK-8 kit was added, incubated for 1 h, and the absorbance of OD450 was measured again.

### 2.10. Culture of Chicken Adipose Tissue and Glycerol Detection

The four-month-old male chickens (Lohmann White Layer) were subjected to the adipose tissue culture. The healthy chickens were decapitated, their abdominal fat tissues were quickly removed and cut into small pieces with a size of about 1 cm^3^. The cut adipose tissue pieces were evenly distributed in 48-well plates and pre-incubated with KRB buffer containing 1% albumin for 1 h. We then replaced the polypeptide diluted with KRB buffer (containing 1% albumin), incubated the samples at 37 °C for 6 h, and shook them manually every half hour. Then the buffer was collected to detect the glycerol content using a glycerol detection kit (Applygen, Beijing, China) according to the manufacturer’s instructions.

### 2.11. Data Analysis

The relative expression level of *SSTR2* mRNA was calculated using the 2^−ΔΔCT^ method with *β-actin* as the internal reference gene [36]. Semi-quantitative analysis of band intensity from Western blot was performed using the Image J software java 1.8.0 (National Institutes of Health, Bethesda, MD, USA), and the relative phosphorylated ERK 1/2 (pERK 1/2) levels normalized by that of intracellular total ERK 1/2 were then expressed as the percentage compared with respective controls (without treatment). The data were analyzed using Student’s *t*-test (between two groups) in GraphPad Prism 7 (GraphPad Software, San Diego, CA, USA). To validate our results, all experiments were repeated at least three times.

## 3. Results

### 3.1. Expression of SSTR1-5 in Chickens

**Figure 1:**
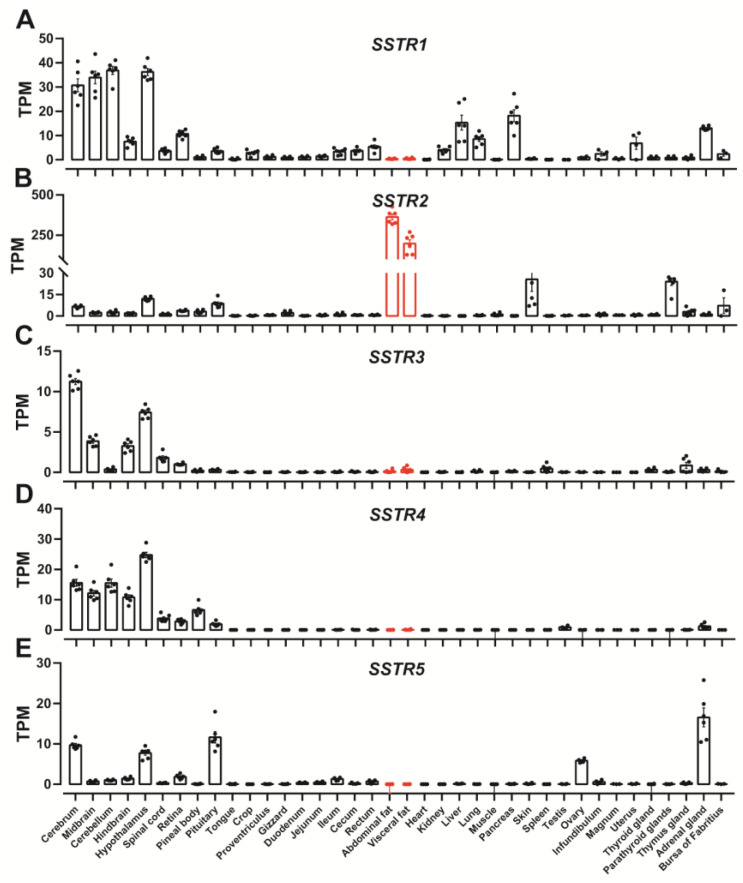
RNA-seq data showing the expression of *SSTR1* (**A**), *SSTR2* (**B**), *SSTR3* (**C**), *SSTR4* (**D**) and *SSTR5* (**E**) in adult Lohmann chickens (CNP0003404). Each data point represents the mean ± SEM of six adult chickens (three males and three females) (*n* = 6). However, uterus, infundibulum, magnum, and ovary represent the mean ± SEM of three adult female chickens (*n* = 3). Similarly, testis represents the mean ± SEM of three male chickens (*n* = 3). Red is used to highlight adipose tissue data.

RNA-seq data were used to investigate the expression abundance of *SSTR1-5* in 38 tissues of Lohmann White chickens. As shown in Figure 1A–E, *SSTR1-5* were widely expressed in the central nervous system of chickens, including the cerebrum, cerebellum, midbrain, hindbrain, hypothalamus and spinal cord. The expression of *SSTR1-5* in the central nervous system was subtype-specific, with high expression levels for *SSTR1* and *SSTR4* (Figure 1A–E). The main somatostatin receptors expressed in peripheral tissues were *SSTR1*, *SSTR2* and *SSTR5*, whereas *SSTR3* and *SSTR4* were hardly expressed (Figure 1A–E). *SSTR1* was expressed in the liver, lung, pancreas, and adrenal gland (Figure 1A). *SSTR5* was expressed in the ovary, pituitary, and adrenal gland (Figure 1E). *SSTR2* was expressed in the pituitary, parathyroid gland, skin, abdominal fat and visceral fat (Figure 1B). Strikingly, the expression level of *SSTR2* was particularly high in adipose tissue and was higher than in all other tissues detected (Figure 1B). Although *SSTR1* and *SSTR3-5* were also expressed in adipose tissue, their TPM values were less than one (Figure 1A,C–E).

### 3.2. Validation of SSTR2 Expression in Chicken Adipose Tissue

**Figure 2:**
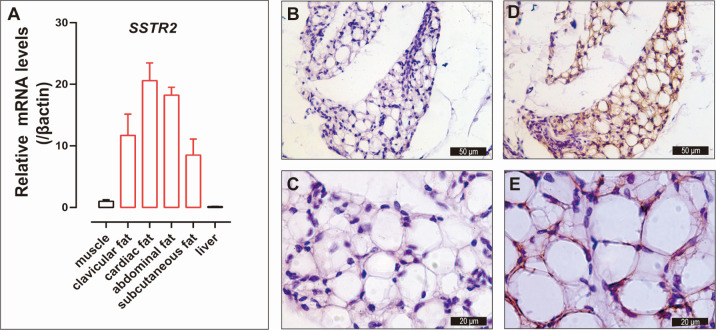
Validation of SSTR2 expression in adipose tissue of Lohmann chickens. (**A**) qPCR analysis of *SSTR2* transcripts in subcutaneous fat, clavicular fat, abdominal fat, cardiac fat, muscle and liver of adult Lohmann chickens. Each data point represents the mean ± SEM of four adult male chickens (*n* = 4). Red is used to highlight adipose tissue data. (**B**–**E**) Immunohistochemical staining of SSTR2 protein in chicken adipose tissues. Negative controls (**B**,**C**) were incubated with primary antibody dilutions without added SSTR2 antibody. Hematoxylin was used to stain the nuclei in both the negative controls and experimental groups. The cell nucleus is blue-purple, and the SSTR2-specific signal is brown.

Adipose tissue is often distributed in several fat depots. Generally, fat depots, such as abdominal fat, which is the largest fat depot, along with fat pads in the neck, thigh, back, and gizzard, collectively constitute approximately 20% of total body fat content [37]. In the present study, the cDNAs from chicken clavicular fat, cardiac fat, abdominal fat, subcutaneous fat, liver, and muscle were isolated for the validation of the expression of *SSTR2*. As shown in Figure 2A, the *SSTR2* showed a high expression abundance in chicken clavicular fat, cardiac fat, abdominal fat and subcutaneous fat but was relatively less expressed in the liver and muscle. In order to determine the expression of SSTR2 protein in chicken adipose tissue, we collected abdominal adipose tissue from Lohmann White chickens for immunohistochemical staining. As shown in Figure 2B–E, immunohistochemical staining results showed obvious SSTR2 positive signals (brown) in the cytoplasm of adipocytes, and SSTR2 was expressed in both multilocular adipocytes and mature unilocular adipocytes.

### 3.3. Expression of SSTR2 in Red Jungle Fowl and J-Line Chickens

**Figure 3:**
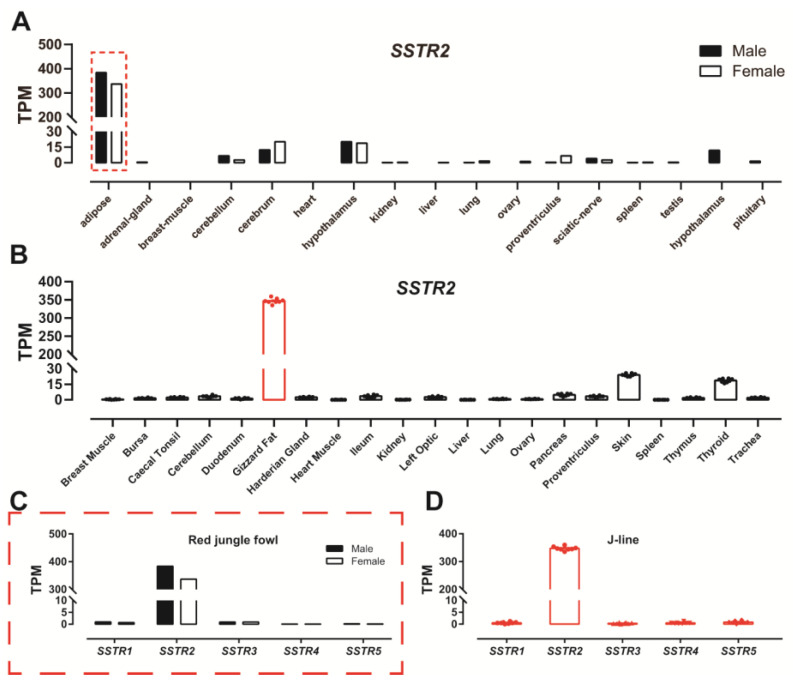
RNA-seq data showing the expression of *SSTR1-5* in red jungle fowls (SRP016501) and J-line chickens (ERP014416). (**A**,**C**) Expression of *SSTR2* in 17 red jungle fowl tissues (**A**) and expression of *SSTR1-5* in red jungle fowl adipose tissue (**C**). Each data point represents one adult female or one adult male (*n* = 1). (**B**,**D**) Expression of *SSTR2* in 21 J-line chicken tissues (**B**) and expression of *SSTR1-5* in J-line chicken adipose tissue (**D**). Each data point represents the mean ± SEM of nine chickens (*n* = 9). Red is used to highlight adipose tissue data.

To determine whether the expression of *SSTR2* in adipose tissue from Lohmann White chickens is strain-specific, we collected transcriptome data of red jungle fowl and J-line chickens. As shown in Figure 3A–D, the expression of *SSTR2* was detected in the adipose tissue of red jungle fowl (Figure 3A,C) and J-line chicken (Figure 3B,D), and the expression levels were consistent with those of Lohmann White chickens. Whether in red jungle fowl (Figure 3C) or J-line chickens (Figure 3D), the expression levels of *SSTR2* in adipose tissue are the highest, whereas the expression levels of *SSTR1* and *SSTR3-5* are extremely low.

### 3.4. The Anti-Lipolytic Effect Mediated by SSTR2 in Chicken Adipose Tissue

**Figure 4:**
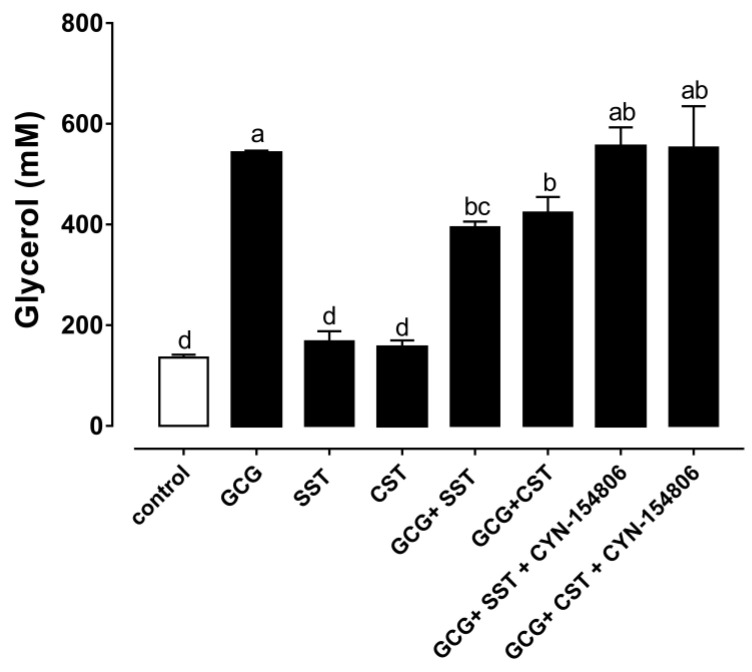
Effects of somatostatin and cortistatin on lipolysis in chicken adipose tissue. CYN-154806 is an SSTR2 antagonist. All peptides are used at a concentration of 100 nM. Each data point represents the mean ± SEM of four replicates (*n* = 4). Significant differences are marked with lowercase letters. No identical lowercase letter between two groups indicates significant differences (*p* < 0.05).

The high abundance of chicken *SSTR2* in chicken adipose tissues led us to further investigate its role in lipid metabolism. The effect of glucagon, SST28, CST14 and CYN-154806 (SSTR2 antagonist) on glycerol release was examined in the cultured chicken adipose tissue. As shown in Figure 4, the GCG treatment induced glycerol release immediately. In contrast, SST28 and CST14 alone did not inhibit the basal glycerol release in the cultured chicken adipose tissue (Figure 4). However, SST28 effectively inhibited the GCG induced glycerol release. Similarly, CST14 also effectively inhibited the GCG-induced glycerol release. As shown in Figure 4, the treatment of CYN-154806, an SSTR2 antagonist, effectively blocked the inhibitory effect of SST28 and CST14 on GCG-stimulated glycerol release.

### 3.5. Activation of SSTR2 Stimulates the Proliferation of ICP-1 Cells

**Figure 5:**
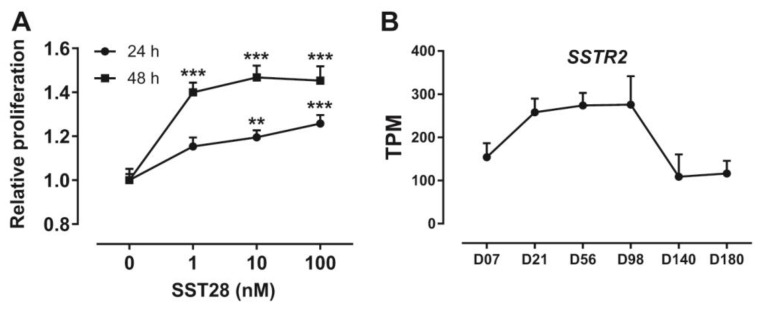
(**A**) The CCK-8 assay showing the effect of SST28 on the proliferation of SSTR2-expressing ICP-1 cells. Each data point represents the mean ± SEM of six replicates (*n* = 6). (**B**) The RNA-Seq data showing the developmental expression of *SSTR2* mRNA in Jingxing-Huang broiler chickens (PRJCA001192). Each data point represents the mean ± SEM of three chickens (*n* = 3). **, *p* < 0.01 vs. 0 nM SST28 treatment, ***, *p* < 0.001 vs. 0 nM SST28 treatment.

Increases in adipocyte number and adipocyte volume are main factors in the development of adipose tissue in animals. In mammals, somatostatin is often reported to have inhibitory effects on cell proliferation. Considering that proliferation is one of the important factors affecting adipose tissue development, we investigated the effect of SSTR2 activation on proliferation in immortalized chicken preadipocytes. As shown in Figure 5A, SST28 treatment dose-dependently stimulated an increase in the number of ICP-1 cells as measured by cell counting kit-8 (CCK-8), whether assayed at 24 h or 48 h.

### 3.6. Developmental Expression Pattern of SSTR2 in Chickens

In order to clarify the expression pattern of *SSTR2* during adipose tissue development after hatching, we analyzed the transcriptome data of abdominal adipose tissue of Jingxing-Huang broilers during the developmental period (PRJCA001192). As shown in Figure 5B, the expression of *SSTR2* rapidly increased during the period Day 7–Day 21, remained stable with high abundance during the stage Day 56–Day 98, and decreased rapidly during Day 98–Day 140.

### 3.7. Identification of SSTR2 Signaling Pathway

**Figure 6:**
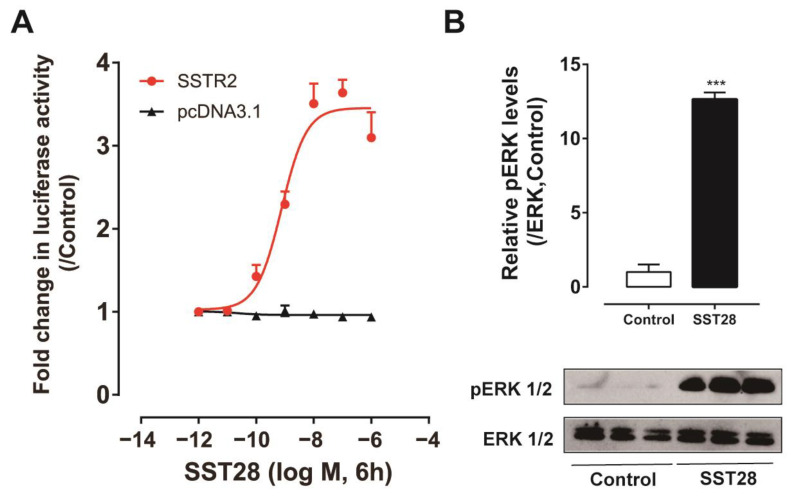
Effect of cSST28 on activating chicken SSTR2 expressed in CHO cells. (**A**) Effect of cSST28 on activating chicken SSTR2 expressed in CHO cells, monitored by pGL4-SRE-luciferase reporter system. CHO cells co-transfected with empty pcDNA3.1 (+) vector and pGL4-SRE-luciferase reporter construct were used as internal controls, and peptide treatment did not alter the luciferase activity of CHO cells at any concentration tested. Each data point represents the mean ± SEM of three replicates (*n* = 3). (**B**) Western blot showing that cSST28 treatment (10 nM, 10 min) can enhance the phosphorylation levels of ERK1/2 (pERK1/2) in CHO cells expressing chicken SSTR2 (cSSTR2). Phosphorylated ERK1/2 (pERK) levels were determined by densitometric analysis and normalized to that of total ERK1/2 and expressed as the fold difference compared with the control. Each data point represents the mean ± SEM of three replicates (*n* = 3). ***, *p* < 0.001 vs. control.

The chicken SSTR2 coupled signaling pathway was further studied through the pGL4-SRE-luciferase reporter system in cultured Chinese hamster ovary (CHO) cells. As shown in Figure 6A, SST28 stimulated luciferase activity in a dose-dependent manner in CHO cells transfected with *cSSTR2* expression vector and pGL4-SRE-luciferase reporter construct, suggesting that cSSTR2 is functionally coupled to Gi/Gq proteins. In the pGL4-SRE-luciferase reporting system, the activation concentration of SST28 is nanomolar. Western blot was further employed to detect the downstream molecule ERK in the MAPK signaling pathway. As shown in Figure 6B, SST28 treatment potently enhanced the phosphorylation of ERK in CHO cells transfected with the cSSTR2 expression vector. The uncropped Western blot membrane is shown in Figures S1 and S2 in the Supplementary Materials. In addition, the cSSTR2 activation resulted in a 10-fold increase in pERK phosphorylation quantified by Image J software java 1.8.0 (Figure 6B).

## 4. Discussion

The present study investigated the expression of *SSTR1-5* in chickens and found that *SSTR2* transcripts were abundantly expressed in the adipose tissue of red jungle fowl, Lohmann White chickens and J-line chickens. SSTR2 protein was also detected in the adipocytes of Lohmann chickens by immunohistochemistry. In the cultured chicken adipose tissue, SSTR2 agonist blocked the inhibition of GCG-stimulated glycerol release by somatostatin, providing direct evidence supporting an SSTR2-mediated anti-lipolytic effect. We also observed that activation of SSTR2 stimulated the proliferation of ICP-1 cells, and this effect may involve the mediation of the intracellular MAPK/ERK signaling pathway. Current research supports that activation of SSTR2 in chicken adipose tissue produces an anti-lipolytic effect and may have a pro-proliferative effect on preadipocytes, thereby actively participating in the metabolism and development regulation of chicken adipose tissue.

### 4.1. SSTR2 Is Expressed in Chicken Adipose Tissue

Although the expression of *SSTR1-5* in various chicken tissue was initially investigated using semi-quantitative PCR, encompassing tissue such as the heart, duodenum, kidneys, lung, muscle, ovary, testes, anterior pituitary, spleen, pancreas, telencephalon, mid-brain, cerebellum, hindbrain, and hypothalamus, transcriptome data analysis offers a more comprehensive approach to quantifying gene expression abundance across different tissues [20]. Transcriptome data analysis revealed that *SSTR2* is abundantly expressed in chicken adipose tissue, including abdominal fat and visceral fat. In contrast, *SSTR1* and *SSTR3-5* showed negligible expression in adipose tissues. The qPCR result further verified the expression of *SSTR2* in different adipose pads (clavicular fat, cardiac fat, abdominal fat and subcutaneous fat) in Lohmann White chickens. The high expression abundance of SSTR2 in chicken adipose tissue was further supported by the transcriptome data of red jungle fowl and J-line chickens, where abundant expression of *SSTR2* was also detected. According to the evolutionary hypothesis, domestic chickens emanated from the red jungle fowl [38,39]. SSTR2 is also expressed in red jungle fowl, indicating that the function of this gene may have been conserved during chicken domestication. Using immunohistochemical staining, the expression of SSTR2 protein in the adipose tissue of Lohmann White chickens was also confirmed. In rats, SSTR2 is mainly expressed in the brain, pituitary, pancreas, and adrenal glands [40,41,42,43,44]. In humans, SSTR2 is expressed in the brain, kidney, stomach, duodenum, ileum and liver, and has also been detected in adipose tissue [45,46,47,48]. To our knowledge, this is the first time that SSTR2 has been found to be highly expressed in chicken adipose tissue.

### 4.2. SSTR2 Mediates the Anti-Lipolytic Effect in Chicken Adipose Tissue

In contrast to the fact that catecholamines play a major lipolytic role in mammals, glucagon is the main lipolytic hormone in birds [37,49,50]. In this study, we investigated the effects of glucagon, SST28, CST14, and CYN-154806 (SSTR2 antagonist) on glycerol release in cultured chicken adipose tissues. By detecting the concentration of glycerol in the incubation solution, GCG significantly stimulated the lipolysis of chicken adipose tissue, which was consistent with previous reports [23,24,51]. SST28 and CST14 alone did not affect the release of glycerol in adipose tissue, indicating that SST28 and CST14 did not affect basal lipolysis. However, both SST28 and CST14 inhibited GCG-stimulated glycerol release, which is similar to the previous observation that SST28 and SST14 can inhibit GCG-stimulated glycerol release in chicken adipocytes [23]. Current results in chicken adipose tissue or adipocytes indicate that SST28, SST14 and CST14 all produce anti-lipolytic effects [23]. Furthermore, this observed anti-lipolytic effect of somatostatin does not appear to be restricted to domestic chickens. In pancreatectomized or hypophysectomized ducks, the infusion of somatostatin elicited a prompt fall in plasma-free fatty acid levels [28].

CYN-154806 is a potent selective antagonist of SSTR2, displaying a sub-nanomolar affinity for rat SSTR2 expressed in CHO cells [52]. In the present study, CYN-154806 blocked the inhibitory effect of somatostatin on GCG-stimulated glycerol release, supporting that the anti-lipolytic effects of somatostatin are mediated through SSTR2. According to the results of a previous experiment, SST28, SST14 and CST14 have the same potency in activating SSTR2 in chickens, which explains why SST28, SST14 and CST14 all produce anti-lipolytic effects [20]. Although CSTs share structural, pharmacological, and functional similarities with SSTs, their coding gene is reported to have evolved from different ancestral genes, indicating that they are not simply natural substitutes for SSTs [6,53]. The present study supports that CST14 may serve as one more anti-lipolytic peptide involving chicken lipid metabolism. In duck peripheral plasma, only the large form of somatostatin (SST28) was found, and this somatostatin-like immunoreactivity concentration was 1.05 ± 0.45 μg/L (*n* = 11, range 0.84–1.2 μg/L) [54]. It is unclear whether the immunoreactivity of somatostatin in the peripheral plasma of chickens is similar to that of ducks. Under physiological conditions, the natural ligand that activates SSTR2 in chicken adipose tissue remains to be identified.

### 4.3. SSTR2 May Stimulate the Proliferation of Chicken Preadipocytes by Activating the MAPK/ERK Signaling Pathway

The development of adipose tissue requires an increase in cell number (hyperplasia) and volume (hypertrophy) [37]. Because the number and volume of adipocytes in chickens increase with age and are positively correlated with the weight of the fat pad, both the hypertrophy and hyperplasia of adipocytes lead to fat accumulation [37,55]. In chicken preadipocytes, SST28 dose-dependently stimulates the proliferation of ICP-1 cells expressing SSTR2, suggesting that somatostatin may stimulate the hyperplasia process of chicken adipocytes. This result is contrary to a previous finding that somatostatin inhibits the proliferation of 3T3-L1 cells [56]. 3T3-L1 is a mouse preadipocyte cell line, and the difference in results between ICP-1 and 3T3-L1 may be due to species differences. The hypertrophy and hyperplasia of adipocytes contribute differently to the development of adipose tissue at different stages. Generally speaking, the growth of fat pads in youth is mainly attributed to an increase in the number of adipocytes, but in later stages, the accumulation of lipids in adipocytes is the main determining factor. In the present study, a survey of *SSTR2* mRNA expression in a chicken commercial broiler line was investigated. As shown in Figure 5B, the expression of *SSTR2* mRNA exhibited a notably high level during the initial 14 weeks, aligning with findings indicating a rise in the number of abdominal adipocytes until approximately 12-14 weeks of age, whereas adipocyte volume experienced gradual growth [37]. Beyond this age, filling of the existing adipocytes is the main factor that contributes to the weight gain of the abdominal fat pad [37,55,57,58]. Our study shows that SSTR2 mainly affects the development of adipose tissue by controlling the hyperplasia of adipocytes before 14 weeks and then by influencing the hypertrophy of adipocytes after 14 weeks.

**Figure 7:**
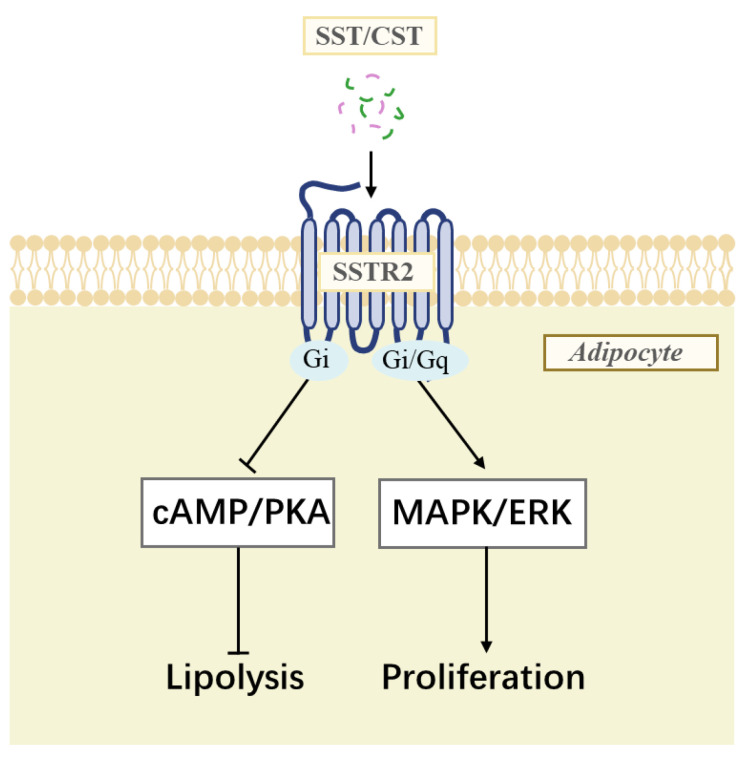
Schematic diagram shows the ligand–receptor interaction of SST/CST and SSTR2 in chicken adipocytes. Both SST and CST can activate SSTR2 expressed on the adipocyte membrane. The coupling of SSTR2 and Gi protein inhibits the cAMP/PKA signaling pathway to produce anti-lipolytic effects. The coupling of SSTR2 and Gi/Gq protein stimulates the MAPK/ERK signaling pathway to promote adipocyte proliferation.

A previous study demonstrated that chicken SSTRs are coupled to Gi proteins, which inhibit the cAMP/PKA signaling pathway [20]. According to the reports, the cAMP/PKA signaling pathway is related to the regulation of lipolysis [59]. The inhibitory effect of somatostatin on GCG-stimulated lipolysis appears to be through the cAMP/PKA signaling pathway. To understand the molecular mechanisms of the SSTR2-mediated proliferation effect, we further investigated the SSTR2 coupled signaling pathway using the luciferase reporter system and Western blot. We found that cSSTR2 strongly activated the MAPK/ERK signaling cascade. The MAPK/ERK signaling pathway is an important regulator of cell proliferation and differentiation in mammals [60,61,62]. Studies have shown that ERK phosphorylation is required for 3T3-L1 to differentiate into adipocytes [63]. The proliferative effect of SST on ICP-1 cells seems to be regulated through the MAPK/ERK signaling pathway. The hypothesized effects of the signaling pathway activated by SSTR2 in chickens are shown in Figure 7.

## 5. Conclusions

In summary, we demonstrated that *SSTR2* was highly expressed in chicken abdominal fat and visceral fat. In the cultured adipose tissue, SST28 and CST14 inhibited GCG-stimulated glycerol release, and an SSTR2 antagonist (CYN-154086) blocked this anti-lipolytic effect induced by somatostatin, supporting that SSTR2 mediated the anti-lipolytic effect of SST/CST in chicken adipose tissue. In addition, we found that the activation of SSTR2 in adipose tissue may stimulate the proliferation of preadipocytes through the MAPK/ERK signaling pathway, and thus may have a dual impact on the development of adipose tissue.

## Data Availability

The supporting data of this study are available within the article.

## Supplementary Materials

The following supporting information can be downloaded at: https://www.mdpi.com/article/10.3390/ani14071034/s1. Figures S1 and S2: Western blot membrane of ERK (~42 kDa) detected with anti-ERK antibodies.
