# Supplementary material for: SSTR2 Mediates the Inhibitory Effect of SST/CST on Lipolysis in Chicken Adipose Tissue
Source: Animals (Basel). 2024 Mar 28;14(7):1034. doi: 10.3390/ani14071034 (PMC11010918; doi:10.3390/ani14071034)
Supplement: Supplementary file 1 [file animals-14-01034-s001.zip › animals-2886812-supplementary.pdf]

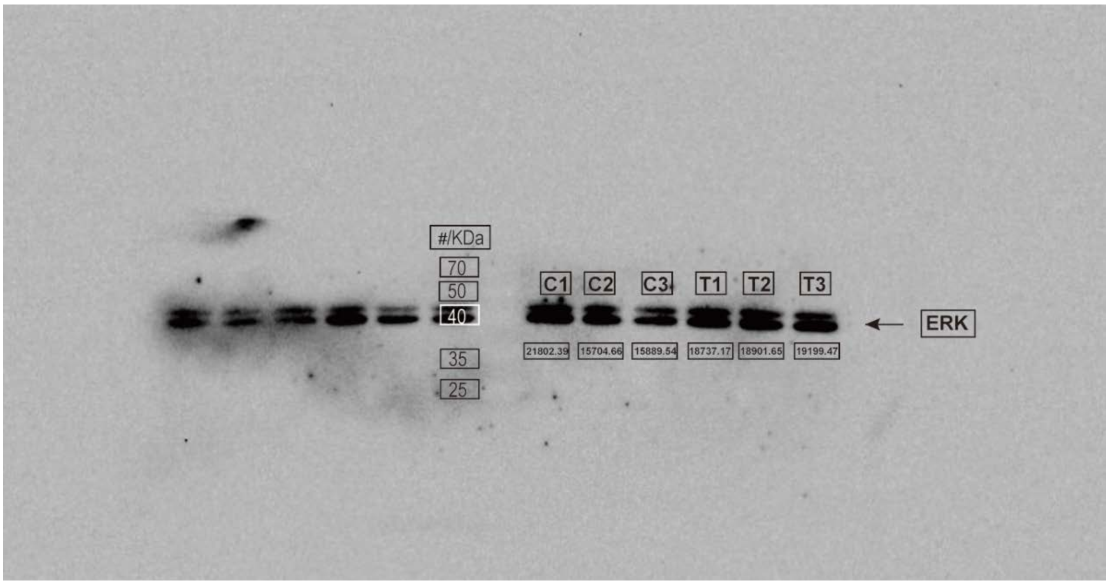

Figure S1. Western blot membrane of ERK (~42 kDa) detected with anti-ERK antibodies. Protein marker of 10-250kDa was used in electrophoresis to estimate the size of target proteins. After cutting the Gel according to the size of the target protein, the separated proteins in the glue were transferred to the polyvinylidene fluoride (PVDF) membrane. PVDF membrane was incubated with anti-ERK primary antibody, and a secondary antibody conjugated with horseradish peroxidase. Finally, the abundance of target proteins was detected using enhanced chemiluminescence substrates on a Bio-rad ChemiDoc Touch instrument. The position of protein markers on the PVDF membrane after exposure were marked on the picture. Before measuring the density, the imprinted image was converted to grayscale using ImageJ software as follow: Image -> Type –> 8 bit. The measured densitometry readings were indicated on the picture.

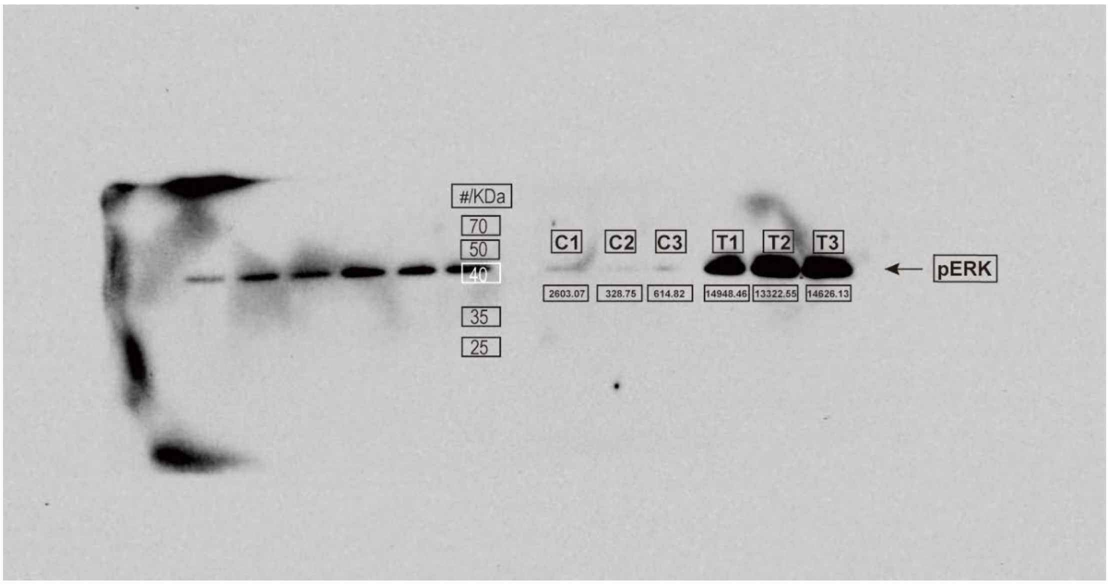

Figure S2. Western blot membrane of ERK (~42 kDa) detected with anti-ERK antibodies. Protein marker of 10-250kDa was used in electrophoresis to estimate the size of target proteins. After cutting the Gel according to the size of the target protein, the separated proteins in the glue were transferred to the polyvinylidene fluoride (PVDF) membrane. PVDF membrane was incubated with anti-ERK primary antibody, and a secondary antibody conjugated with horseradish peroxidase. Finally, the abundance of target proteins was detected using enhanced chemiluminescence substrates on a Bio-rad ChemiDoc Touch instrument. The position of protein markers on the PVDF membrane after exposure were marked on the picture. Before measuring the density, the imprinted image was converted to grayscale using ImageJ software as follow: Image -> Type –> 8 bit. The measured densitometry readings were indicated on the picture.
